# The Neurokinin-1 Receptor Is Essential for the Viability of Human Glioma Cells: A Possible Target for Treating Glioblastoma

**DOI:** 10.1155/2022/6291504

**Published:** 2022-04-04

**Authors:** Mario F. Muñoz, Sandro Argüelles, Marisa Rosso, Rafael Medina, Rafael Coveñas, Antonio Ayala, Miguel Muñoz

**Affiliations:** ^1^Department of Biochemistry and Molecular Biology, University of Seville, Seville, Spain; ^2^Department of Physiology, University of Seville, Seville, Spain; ^3^Virgen del Rocío, University Hospital, Research Laboratory on Neuropeptides (IBIS), Seville, Spain; ^4^Institute of Neurosciences of Castilla Y León (INCYL), Laboratory of Neuroanatomy of the Peptidergic Systems, University of Salamanca, Salamanca, Spain

## Abstract

**Background:**

Glioblastoma or glioma is the most common malignant brain tumor. Patients have a prognosis of approximately 15 months, despite the current aggressive treatment. Neurokinin-1 receptor (NK-1R) occurs naturally in human glioma, and it is necessary for the tumor development.

**Objective:**

The purpose of the study was to increase the knowledge about the involvement of the substance P (SP)/NK-1R system in human glioma.

**Methods:**

Cellular localization of NK-1R and SP was studied in GAMG and U-87 MG glioma cell lines by immunofluorescence. The contribution of both SP and NK-1R to the viability of these cells was also assessed after applying the tachykinin 1 receptor (*TAC1R*) or the tachykinin 1 (*TAC1*) small interfering RNA gene silencing method, respectively.

**Results:**

Both SP and the NK-1R (full-length and truncated isoforms) were localized in the nucleus and cytoplasm of GAMG and U-87 MG glioma cells. The presence of full-length NK-1R isoform was mainly observed in the nucleus, while the level of truncated isoform was higher in the cytoplasm. Cell proliferation was decreased when glioma cells were transfected with *TAC1R* siRNA, but not with *TAC1*. U-87 MG cells were more sensitive to the effect of the *TAC1R* inhibition than GAMG cells. The decrease in the number of glioma cells after silencing of the *TAC1R* siRNA gene was due to apoptotic and necrotic mechanisms. In human primary fibroblast cultured cells, *TAC1R* silencing by siRNA did not produce any change in cell viability.

**Conclusions:**

Our results show for the first time that the expression of the *TAC1R gene* (NK-1R) is essential for the viability of GAMG and U-87 MG glioma cells. On the contrary, the *TAC1R gene* is not essential for the viability of normal cells, confirming that NK-1R could be a promising and specific therapeutic target for the treatment of glioma.

## 1. Introduction

Glioblastoma (GB) or glioma (WHO grade IV) is the most common and lethal brain tumor [1], occurring in 90% of patients [2]. Several therapeutic strategies have been developed for the treatment of glioma (e.g., Stupp protocol, treatment with bevacizumab, microRNA, immunotherapy, gene therapy, and intranasal drug delivery) [1, 3], but unfortunately, the prognosis is very poor with approximately 15 months of median survival time [4]. Therefore, it is necessary to investigate the mechanisms underlying the development of glioma and the seeking of new therapeutic strategies to target this tumor.

One of these plausible mechanisms could be related to the substance P (SP)/neurokinin-1 receptor (NK-1R) system. Many studies have shown that SP and NK-1R are involved in the growth and development of different tumors (including malignant hematopoietic cells) [5–7]. SP, via the NK-1R, plays a key role in all stages of tumor development such as cell proliferation/migration, invasion, and metastasis and also exerts an antiapoptotic effect in cancer cells [7–10]. Tumor cells also express NK-1R and SP, suggesting that SP exerts an autocrine action, promoting mitogenesis in cancer cells [6, 11–15]. In melanoma, acute lymphoblastic leukemia, lung cancer, and breast cancer, NK-1R is involved in the viability of human tumor cells [15–17]. NK-1R antagonists have been shown to promote apoptotic mechanisms in tumor cells after binding its receptor [3, 8, 9, 18].

Many studies have demonstrated that the SP/NK-1R system is involved in glioma. Human glioma cell lines [8, 19–22] and glioma samples are extensively described to overexpress NK-1R with respect to normal cells [23]. In these studies, the cellular location of the NK-1R was not reported. SP promotes the proliferation of human glioma cell lines through NK-1R activation [24, 25]. NK-1R antagonists (e.g., L-733,060, aprepitant) inhibit the proliferation in a dose-dependent manner and promote the apoptosis of glioma cells [9, 18, 26, 27]. In GB, the inhibition of NK-1R by L-733,060 decreased the basal kinase activity of Akt, increased the expression of cell cycle regulatory proteins (e.g., p21 and p27), and induced G1/S cell cycle arrest and programmed cell death [28]. A remarkable antitumor synergy between ritonavir and aprepitant was also reported against human glioma cells [3, 9, 26]. Taken together, the data show that NK-1R could be a new therapeutic target against glioma. In this sense, SP/NK-1R system has been previously targeted in radionuclide tumor therapy [29, 30], and the efficacy (e.g., inducing late apoptosis pathways) of targeted alpha therapy with ^213^Bi-DOTA-SP against low-grade glioma and secondary/recurrent GB has been confirmed in patients [31–33].

To increase the knowledge about the involvement of the SP/NK-1R system in glioma and to develop new antiglioma therapeutic strategies, we have studied the cellular localization and expression of NK-1R and SP in human GAMG and U-87 MG glioma cell lines and the involvement of NK-1R in the viability of these cell lines.

## 2. Materials and Methods

### 2.1. Cell Cultures

Three different cell lines were used for this study, two different human tumoral glioma cell lines (GAMG and U87) and a normal human somatic cell (fibroblasts). The human GAMG glioma cell line was obtained from Deutsche Sammlungvon Mikroorganismen und Zellkulturen (DSMZ) (Braunschweig, Germany). The human U-87 MG glioma cell line was generously supplied by Dr. Nelofer Syed (John Fulcher Neuro-Oncology Laboratory, Imperial College London, UK). Skin fibroblasts were obtained from anonymous donors. Both glioma cell lines were cultured in Dulbecco's Modified Eagle's Medium (DMEM) supplemented with 10% heat-inactivated fetal bovine serum (FBS), 2 mM glutamine (Glut), and 1% of penicillin/streptomycin (P/S) maintained in standard culture conditions (37°C in a 5% CO_2_ atmosphere, refreshing the medium every two days). Similar conditions were applied to human fibroblasts except the culture medium that was RPMI/F10 Ham medium (mixture at equal parts 1 : 1), supplemented with 10% FBS, 2 mM glutamine, and 10 mM HEPES, 2% of Ultroser G (PALL Life Science, NY, USA) and 1% of P/S.

### 2.2. Small Interfering (Si)RNA Gene Silencing Method

This method was carried out according to the manufacturer's instructions (Life Technologies, Madrid, Spain). 3 x 10^5^ GAMG and 2.5 x 10^5^ U-87 MG cells were seeded per well in 6-well plates containing 1,750 *μ*l of DMEM supplemented with 1% fetal bovine serum and 2 mM glutamine. 250 *μ*l of transfection reagent medium (20 nM, 10 nM or 5 nM of *TAC1R* siRNA or *TAC1* siRNA) (Invitrogen, Madrid, Spain) and 8 *μ*l of Hiperfect reagent (Invitrogen, Madrid, Spain) were added to each well and incubated for 30 min. After 8 h of incubation, cells were refreshed with 2 ml of normal growth medium. Sham group was also performed, but *TAC1R* siRNA or *TAC1* siRNA were omitted.

### 2.3. Image Acquisition and Analysis

The bright-field images were randomly acquired at different locations using a Nikon Digital Sight camera attached to a Nikon Eclipse TS100 microscope with 20× objective. Three independent experiments were performed. At least two bright-field images were taken for each well. Cells were manually counted and converted to cells/mm^2^.

### 2.4. Western Blot and Subcellular Fractionation

Western blot and subcellular fractionation methods are based on previously optimized protocols by our group [34, 35]. Briefly, cell lines were lysed in RIPA buffer, and protein samples were separated by SDS-PAGE (10% acrylamide) and transferred to a nitrocellulose membrane (Bio-Rad, USA) at 60 V for 2 h. Membranes were incubated with anti-NK-1R antibody (S-8,305, Sigma-Aldrich, Madrid, Spain), diluted 1/1,000, at 4°C. The secondary antibody was a peroxidase-conjugated antirabbit immunoglobulin. Bands were analyzed by densitometry using the ImageJ analysis software (NIH).

For subcellular fractionation, cells were treated as previously described [35]. The cytoplasm and nuclear fractions were processed as previously described for Western blot analysis. Immunoblot was against anti-NK-1R antibody (1/1000), nuclear marker hnRNP (1: 2000) (Santa Cruz Biotechnology, Santa Cruz, CA, USA), and cytoplasmic marker *α*-tubulin (1/1000) (Abcam, Cambridge, MA, USA).

### 2.5. Immunofluorescence

Cells were cultured on coverslips in a 12-well plate. At 80% confluence, the culture medium was removed and washed twice with PBS. Cells were fixed in 4% paraformaldehyde at 4° C for 20 min. The coverslips were washed with PBS and then blocked with 1% fetal bovine serum in PBS for 1 h at room temperature. The coverslips were incubated with rabbit-derived anti-NK-1R antibody (Sigma-Aldrich, Madrid, Spain; 1 : 1,000) or rabbit-derived anti-SP antibody (Sigma-Aldrich, Madrid, Spain; 1 : 1,000) diluted in blocking solution containing 10% serum and 0.25% Triton X-100 in a humid chamber overnight (4°C). Antirabbit Alexa Fluor 488 or Alexa Fluor 647 conjugated (Thermo Fisher, Madrid Spain; 1/500) and Hoechst (Sigma-Aldrich, Madrid, Spain; 1/2000) antibodies were used as secondary incubation. The coverslips were finally washed 3 times and mounted with fluorescent mounting medium (Dako Colorado Inc., Fort Collins, USA). The images were acquired in a confocal microscope (Zeiss LSM7 DUO).

### 2.6. Analysis of Cell Viability and Apoptosis by Flow Cytometer

After silencing of *TAC1R* with *TAC1R*-targeted siRNA in human GAMG and U-87 MG glioma cell lines, viability and apoptosis were determined by flow cytometer (FC500, Beckman Coulter, Pasadena, CA, USA), using Annexin V conjugated with fluorescein isothiocyanate (FITC) and propidium iodide (PI) kit according to the manufacturer's instructions (Miltenyi Biotec, Bergisch Gladbach, Germany). Viable cells (Annexin V^−^, PI^−^) were distinguish from cells in early apoptosis (Annexin V^+^, PI^−^), late apoptosis (Annexin V^+^, PI^+^), and necrosis (Annexin V^−^, PI^+^).

### 2.7. Statistical Analyses

Data are presented as the mean ± SEM. For statistical analysis, the GraphPadPrism Version 5.03 (Graph Pad Software) was used. Statistical evaluation was performed by one-way ANOVA, followed by Tukey's test. A *p* value of ≤0.05 was considered statistically significant.

## 3. Results

### 3.1. Localization of SP/NK-1R in Human GAMG and U-87 Glioma Cell Lines

**Figure 1:**
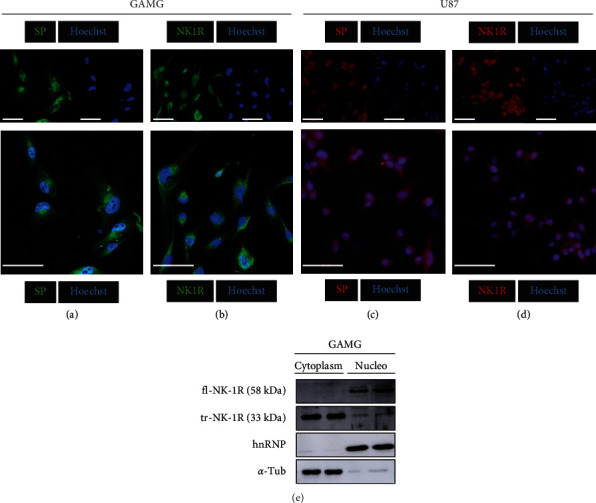
Subcellular localization of SP and NK-1R in human GAMG and U-87 glioma cell lines. Immunofluorescence of SP (a) and NK-1R (b) in GAMG glioma cells and U-87 glioma cells ((c) and (d)). Localization of SP and NK-1R (red) and nuclei (blue). Scale bar =50 *μ*m. (e) Subcellular localization of the NK-1R in GAMG glioma cells by immunoblot. Subcellular fractionation and immunoblot analysis of NK-1R, *α*-tubulin (cytoplasmic marker), and hnRNP (nuclear marker) were performed as described in Materials and Methods.

The presence and localization of SP and NK-1R were studied in human glioma cell lines by immunofluorescence (GAMG and U-87 MG cell lines) and Western blot (GAMG cell line) (Figure 1). Both cell lines expressed SP (Figures 1(a) and 1(c)) and NK-1R (Figures 1(b) and 1(d)). Confocal images confirmed that SP and NK-1R were located in both the nucleus and the cytoplasm. Negative control staining (omitting primary antibodies) did not show evidence of unspecified staining (Supplementary Figure 1). Immunoblot analysis confirmed the subcellular location of two isoforms (full and truncated) of NK-1R in the nucleus and cytoplasm of GAMG glioma cells. The full-length 58 kDa NK-1R isoform (fl-NK-1R) was observed mainly in the nucleus, while the level of the 33 kDa truncated NK-1R isoform (tr-NK-1R) was higher in the cytoplasm (Figure 1(e)).

### 3.2. Effect of *TAC1R*/*TAC1* Gene Silencing by siRNA on the Proliferation of Human GAMG and U-87 MG Glioma Cell Lines

**Figure 2:**
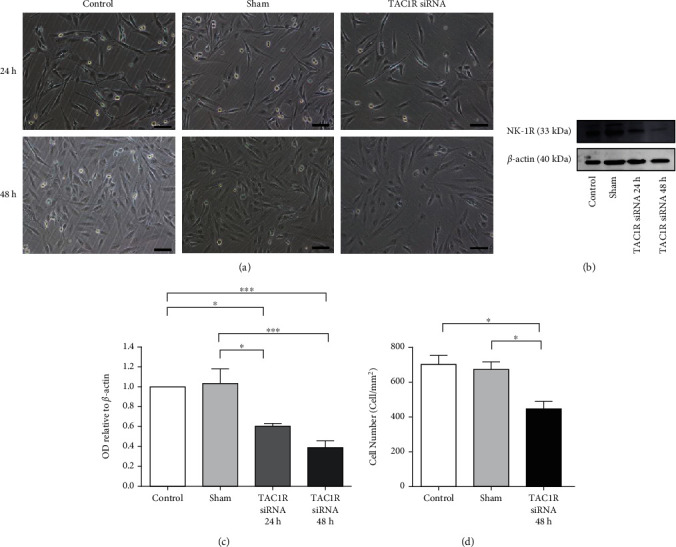
NK-1R expression in *TAC1R* silenced by siRNA in GAMG glioma cells. (a) Images from glioma cultures at 24 h and 48 h. (b) NK-1R and *β*-actin immunoblot. (c) Immunoblot analysis after measuring the OD bands relative to *β*-actin. Data are shown as the mean ± SEM of three independent experiments (*n* = 3 per group). (d) Cell counting after 48 h of treatment were performed as described in Materials and Methods. ∗*p* < 0.05; ∗∗*p* < 0.01.

To study the role of the NK-1R and SP in human GAMG and U-87 MG glioma cells, *TAC1R* and *TAC1* were depleted in these cells using siRNA. *TAC1R* siRNA transfection did not produce any abnormality in the phenotype of GAMG glioma cells (Figure 2(a)). However, a significant decrease in NK-1R expression was observed by Western blot (Figure 2(b)) at 24 h and 48 h, showing significant suppression effects compared to control and sham groups. This decline was time-dependent (Figure 2(c)). NK-1R depletion produced a significant decrease in cell proliferation at 48 h after transfection of GAMG glioma cells with si*TAC1R*. The cell number in *TAC1R* siRNA group was significantly lower (446.8 ± 43.0 cell/mm^2^) than control (702.1 ± 52.5 cell/mm^2^) and sham (673.8 ± 43.1 cell/mm^2^) groups (Figure 2(d)). By contrast, the silencing of *TAC1* expression by *TAC1* siRNA did not produce any change in cell proliferation in GAMG glioma cells (data not shown). Thus, the inhibition of SP expression did not affect the survival of these cells.

**Figure 3:**
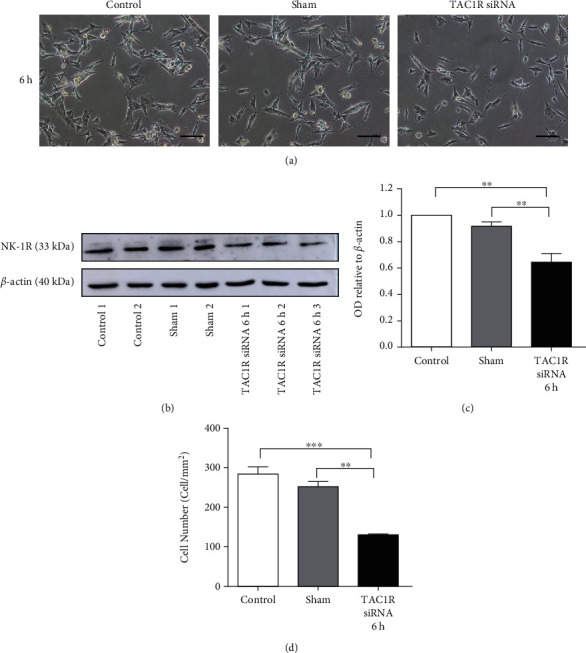
NK-1R expression in s *TAC1R* silenced by siRNA in U-87 glioma cells. (a) Images from glioma cultures at 6 h with 10 nM of siTAC1R. (b) NK-1R and *β*-actin immunoblot. (c) Immunoblot analysis after measuring the OD bands relative to *β*-actin. Data are shown as the mean ± SEM of three independent experiments (*n* = 3 per group). (d) Cell counting after 6 h of treatment was performed as described in Materials and Methods. ∗∗*p* < 0.01; ∗∗∗*p* < 0.001.

**Figure 4:**
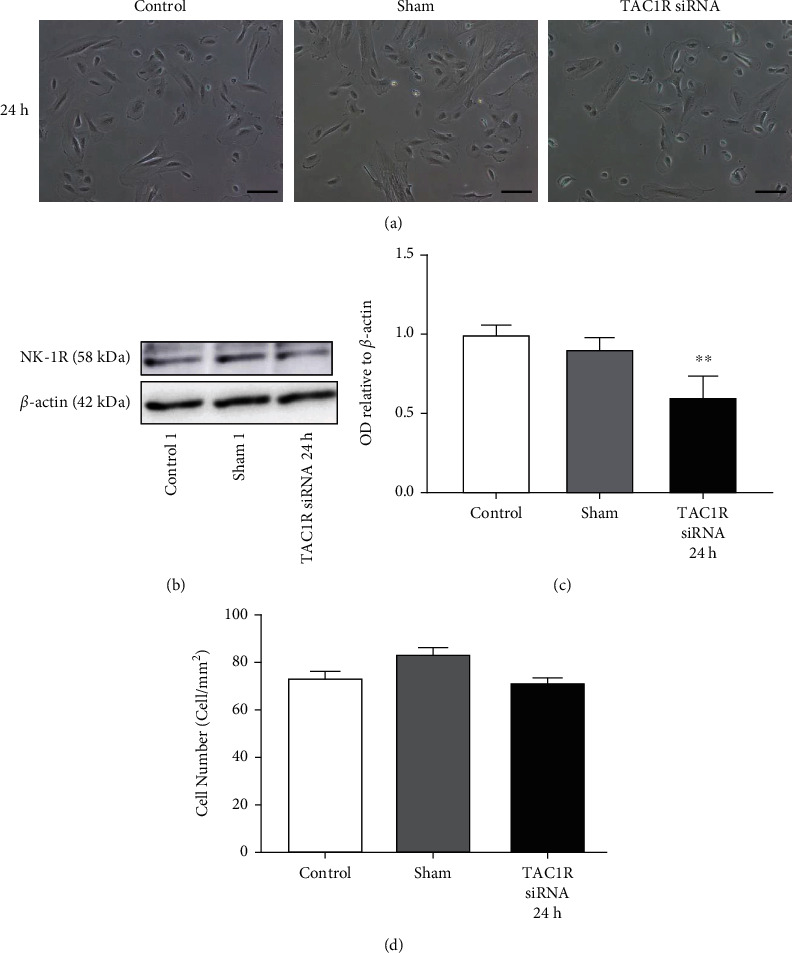
NK-1R expression in *TAC1R* silenced by siRNA in human fibroblast cells. (a) Images from human fibroblast cultures at 24 h with 10 nM of siTAC1R. (b) NK-1R and *β*-actin immunoblot. (c) Immunoblot analysis after measuring the OD bands relative to *β*-actin. Data are shown as the mean ± SEM of three independent experiments (*n* = 3 per group). (d) Cell counting after 24 h of treatment was performed as described in Materials and Methods. ∗∗*p* < 0.01.

Inhibition of the *TAC1R* expression in U-87 MG human glioma cell also leads to time-dependent reduction in the expression levels of the NK-1R (Figures 3(a)–3(c)). *TAC1R* siRNA concentration and treatment duration was optimized to 10 nM during 6 h. Importantly, depletion of *TAC1R* resulted in a significantly cell proliferation decrease (48.18%) as showed the cell count number (cell/mm^2^) at 6 h compared with control cells (Figure 3(d)). 5 nM of *TAC1R* siRNA was also tested, but no significant difference between the control and treated groups was observed (Supplementary Figure 2). These results showed that the U-87 MG cell line was more sensitive to the effect of the *TAC1R* inhibition than GAMG cell line. Unlike, no detrimental effect of silencing of *TAC1R* gene by siRNA was observed in the proliferation of human fibroblast cell culture (Figure 4).

### 3.3. Depletion of the NK-1R Reduces Cell Viability in Human Glioma Cell Lines

**Figure 5:**
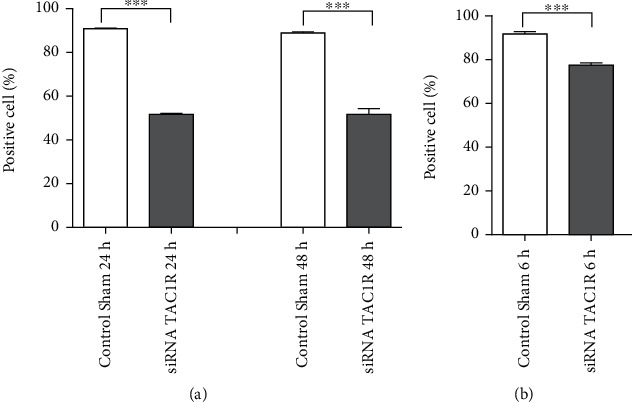
Depletion of NK-1R by TAC1R-targeted siRNA decreases cell viability in human glioma cells. GAMG cells were treated with 20 nM of siRNA for 24 h and 48 h (a), and U-87 glioma cells were treated with 10 nM of siRNA for 6 h (b). Then, cell viability was measured by flow cytometry using Annexin V–FITC and propidium iodide (PI) as described in Materials and Methods. Values are the means ± SEM of three experiments. ∗∗∗*p* < 0.001.

To investigate the role of the NK-1R on cell survival, the effect of its silencing in GAMG and U-87 MG glioma cells was analyzed. NK-1R-depleted cells showed a significantly decreased cell viability compared to control sham. There was observed a decrease of 48.4% and 51% at 24 h and 48 h, respectively, in GAMG (Figure 5(a)), and 22.6% at 6 h in U-87 MG cells (Figure 5(a)).

### 3.4. Depletion of the NK-1R Induces Both Apoptosis and Necrosis in Human Glioma Cells

**Figure 6:**
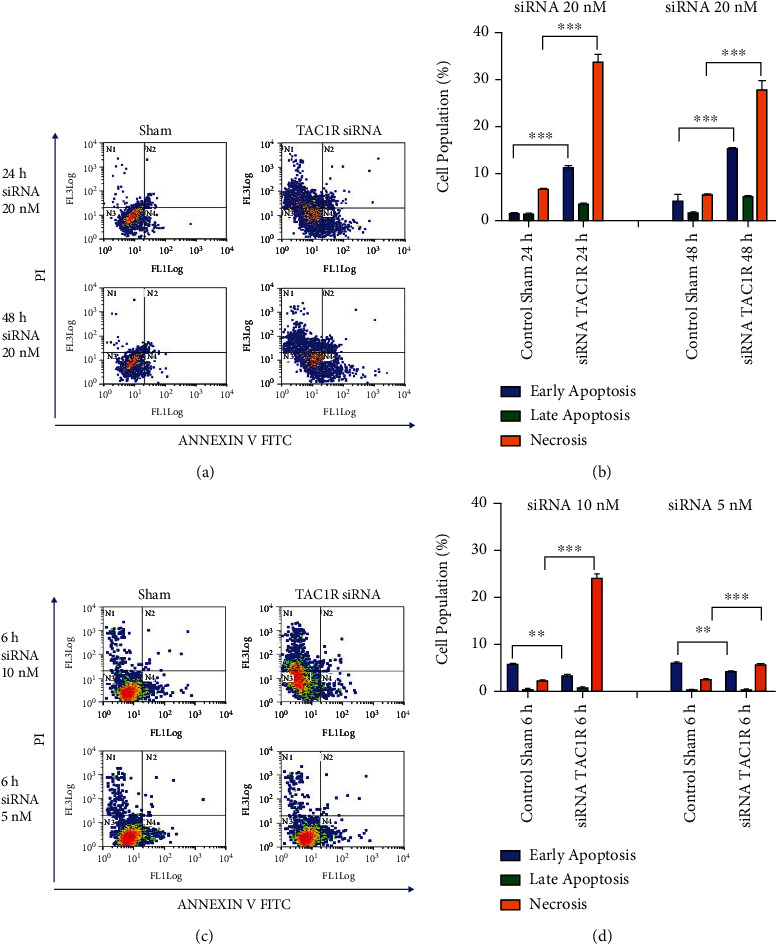
Depletion of NK-1R by TAC1R-targeted siRNA induced both apoptotic and necrotic cell death in human glioma cells. (a) Flow cytometry analysis using Annexin V–FITC and propidium iodide (PI) as described in Materials and Methods was performed to detect cell death in GAMG glioma cells after siRNA transfection at 24 h and 48 h. (b). Cell death rate in GAMG glioma cells. (c). Flow cytometry analysis was used to detect cell death in U87 glioma cells after siRNA transfection at 6 h. (d). Cell death rate in U-87 glioma cells treated with 10 nM and 5 nM of siRNA. Values are the means ± SEM of three experiments. ∗∗*p* < 0.01; ∗∗∗*p* < 0.001.

To study whether the decrease in the number of human glioma cell lines after *TAC1R* siRNA gene silencing was due to an inhibition of the cell proliferation or to an induction of apoptotic or necrotic cell death mechanisms, the number of apoptotic and necrotic cells after transfection by flow cytometer using an Annexin V- conjugated with fluorescein isothiocyanate (FITC) kit was assessed. Viable cells (Annexin V-, PI-) were distinguished from cells in early apoptosis (Annexin V+, PI-), late apoptosis (Annexin V+, PI+), and necrosis (Annexin V-, PI+) stage. Depletion of the NK-1R in GAMG glioma cells led to an increase in both early apoptotic and necrosis population (Figure 6(a)). At 24 h, the early apoptotic positive cells were up to 8.6-fold higher. Necrotic positive cells were up to 5.1-fold higher in NK-1R-depleted cells compared to control sham (Figure 6(b)). In the case of 48 h, while the increase of necrotic positive cells was similar to 24 h (5.1-fold), the early apoptotic positive cells increased only 3.7-fold (Figure 6(b)). In NK-1R-depleted U-87 MG glioma cells (10 nM of TAC1R siRNA), the population of early apoptotic positive cells was up to 1.8-fold in control sham compared to NK-1R-depleted cells, and the necrotic positive cells were up to 11.9-fold higher in NK-1R-depleted cells compared to control sham. When 5 nM of TAC1R siRNA was used, necrotic positive cells were only 2.25-fold higher in NK-1R-depleted cells compared to control sham.

## 4. Discussion

The present findings provide evidence about the antitumor activity of NK-1R gene silencing in human GAMG and U-87 MG glioma cells. We also show for the first time the immunolocalization of SP and NK-1R and the involvement of the NK-1R in the viability of these cells. The fl-NK-1R was mainly observed in the nucleus and the tr-NK-1R isoform in the cytoplasm. U-87 MG cells were more sensitive to the effect of the *TAC1R* inhibition than the GAMG cells. It has been also shown that the *TAC1* gene expression is not relevant for the viability of GAMG glioma cells. Moreover, another interesting finding is that in human nontumor normal fibroblast cells, *TAC1R* silencing by siRNA did not produce any change in cell viability.

The presence of SP and NK-1R in the nucleus and cytoplasm of GAMG and U-87 MG glioma cells is in agreement with previous data reporting the localization of the peptide in the cytoplasm/nucleus of other human cancer cells [7, 14, 15, 23]. These findings confirm that, in general, SP is mainly located in the nucleus and the NK-1R in the cytoplasm of human tumor cells. It is important to mention that here, and in most of the previous studies, the NK-1R was observed in the cytoplasm of tumor cells [7, 14]. The reason is currently unknown, since these receptors are G-protein coupled transmembrane receptors located in the cytoplasmic membrane [36–38]. However, in human primary melanoma, the presence of NK-1R has been found in the cytoplasm and cell membrane of tumor cells [39, 40]. Also, in human primary tumor cells, the NK-1R has been located by immunohistochemistry in the cytoplasm (in all patients studied), cell membrane (80% of patients), and nucleus (46.6%) [15]. Moreover, the number of NK-1Rs in primary tumors of glioma has been reported to be higher than in glioma cultures [3, 41]. It is possible that the immunohistochemical technique applied here in cultured cells was not sensitive enough to detect a clear labeling in the cell membrane and/or the labeling of the cell membrane could be masked by the high labeling for the NK-1R observed in the cytoplasm.

NK-1R exists in two isoforms, as a full-length form (fl-NK-1R) and in the truncated isoform (tr-NK-1R). There are several functional differences between the 2 isoforms depending on the truncation of the 96 C-terminal tail of NK-1R. In our study, the most important finding regarding the localization of the NK-1R is that both the fl-NK-1R and tr-NK-1R isoforms were observed in the cytoplasm (mainly the tr-NK-1R isoform) and nucleus (mainly the fl-NK-1R) of glioma cells. Our results are also in agreement with those observed in adipose stem cells [35].

Internalization of NK-1R on the cell surface is a mechanism that occurs after ligand binding. This could reduce the number of cell surface receptors and thus participate in the desensitization of the response to SP. The absence of the C-terminal tail inhibits the receptor internalization and recycling processes, resulting in both a receptor resistant to desensitization and a longer response after SP binding [27, 42, 43]. Our study shows how the truncated isoform is also located inside the tumor cell like in the fl-NK-1R.

In cancer cells, the expression of the fl-NK-1R isoform is less expressed, meanwhile tr-NK-1R form is overexpressed. Therefore, the expression of the tr-NK-1R isoform, but not the fl-NK-1R, has been related to an enhanced malignant potential (e.g., Ki67 expression and tumor stage) [44]. The fl-NK-1R isoform promoted a slow growth of cancer cells, whereas the tr-NK-1R form increased the growth of tumor cells, mediated malignancy in cancer cells, and stimulated the synthesis of cytokines which exert growth-promoting actions [21]. These cytokines activated the transcription factor NF-*κ*B that upregulated the truncated isoform and slightly increased the full-length isoform [45, 46]. It has been described that tumor cells overexpress miR-206 which regulates the expression of the fl-NK-1R isoform by directly binding the 3′-unstraslated regions of the fl-NK-1R messenger RNA. Overexpression of miR-206 contributes to the malignant phenotype of tumor cells by maintaining a low level of the fl-NK-1R isoform [47]. We describe here that glioma cell proliferation was decreased when glioma cells were transfected by *TAC1R* siRNA. By contrast, in human nontumor normal fibroblast cells, *TAC1R* silencing by siRNA did not produce any change in cell viability. This means that overexpression and continued activation of tr-NK-1R are pivotal for viability of glioma cells. However, less expression and discontinued activation of fl-NK-1R are not pivotal for viability of nontumor cells. This is in agreement with previous results indicating that the expression of NK-1R in human fibroblast cell was much lower than in tumor cells [48]. Moreover, *TAC1R* knockout mice are completely viable and healthy, showing only some cognitive alterations as hyperactivity [49].

An important question is why NK-1R and SP are located in the nucleus of cancer cells. According to previous studies, it seems that SP, via the NK-1R, could regulate the nuclear function and could act as an epigenetic factor in human cancer cells. Thus, in GAMG and U-87 MG glioma cells, the SP/NK-1R system (e.g., via a SP-NK-1R complex) could exert an important role in the regulation of DNA expression (intracrine or nucleocrine action) through the modulation of proto-oncogenes and transcription factors (e.g., AP-1, c-myc, c-jun, c-fos, and hypoxia-inducible factor) involved in apoptosis, in cellular differentiation/transformation, and in cell cycle progression [41, 50].

The presence of SP in the cytoplasm of GAMG and U-87 MG glioma cells is also important since the secretion of SP, through the NK-1R, could display (1) an autocrine action, in which SP exerts a mitogenic action on the own glioma cell from which the peptide was released [51]; (2) a paracrine action, in which SP promotes the proliferation of glioma and endothelial cells located in the vicinity of the glioma cell from which the peptide was secreted; and/or (3) an endocrine action, in which the release of SP from the tumor mass into the blood vessels increases the plasma level of the peptide, reaching the entire body through the bloodstream [25, 52, 53]. In fact, it is important to note that in recent studies carried out in patients with cancer, both the serum SP level and the number of NK-1Rs were higher when compared with healthy controls [15, 53]. Thus, an increased level of SP in serum could be a predictive factor indicating a tumor development and/or a high risk to develop cancer.

It has been reported that SP, after binding to the NK-1R, activates the mammalian target of rapamycin (mTOR) signaling axis in cancer cells and enhances tumor cell growth and metastasis [25]. NK-1R antagonists (e.g., aprepitant) attenuates mTOR activation by reducing the phosphorylation of its downstream effectors (e.g., p70 S6 kinase) [54]. Moreover, SP released from tumor cells or from other sources (e.g., nerve terminals, immune cells) could also promote the migration of tumor cells [55]. SP also promotes the expression of degradative enzymes (matrix metalloproteinases) favoring tumor cell migration, invasion, and metastasis [43, 56], whereas NK-1R antagonists block tumor cell proliferation, migration, and invasion [55]. The activation of NF-*κ*B by SP increased the NK-1R transcription by binding to the *TAC1R* gene [46]. Moreover, it is known that SP, via the NK-1R located in the endothelial cells (placed within the tumor and in the peritumor region), promoted angiogenesis which is crucial for the development of tumors by increasing the tumoral blood supply [23, 57]. Thus, glioma cells by releasing SP could promote angiogenesis, favoring the development of the tumor. Altogether, these findings show the essential role that the NK-1R plays in cancer, confirming that this receptor is an excellent target for the treatment of tumors.

Regarding the potential development of antiglioma strategies, the main finding of this work is that the NK-1R is necessary for the survival of human GAMG and U-87 MG glioma cells. The suppression of the NK-1R expression in these cells, by a knockdown gene silencing method, induced a decrease (mainly due to a dual apoptotic and necrotic mechanism) in the number of GAMG glioma cells. Similar results were observed in U-87 MG glioma cells, although these cells were more sensitive to the effect of the *TAC1R* inhibition than GAMG cells. The reason is currently unknown, but it could be related to the number of the NK-1Rs expressed by glioma cells and, in particular, to the number of full-length/truncated expressed isoforms. Therefore, further studies are required to elucidate the different *TAC1R* inhibition responses between glioma cell lines. Our findings agree with previous studies showing the essential involvement of the NK-1R in the viability of other human tumor cells (melanoma, acute lymphoblastic leukemia, breast cancer, and lung cancer) [15, 47]. This means that NK-1R is crucial for tumor cell survival, and a common antitumor strategy could be applied to treat any tumor. In these previous studies and after applying the knockdown gene silencing method, the technique to demonstrate necrotic mechanisms was not performed, and in all cases, the death of tumor cells was reported to be due to apoptotic mechanisms [3, 15, 18, 26, 58]. In cancer cells, NK-1R silencing promoted G2/M phase arrest/apoptosis and suppressed the proliferation of these cells; similar results were found when the NK-1R antagonist aprepitant was administered, but SP rescued the effects of the NK-1R silencing regarding apoptosis and cell proliferation [44]. Here, our study shows that glioma cells died by necrotic and apoptotic mechanisms. It seems that the absence of the NK-1R in glioma cells induces an acute and irreversible lesion, derived from a nonphysiological situation which produces the breakage of the cell membrane causing the death of glioma cells by necrotic mechanisms.

It is known that SP exerts a mitogenic action in tumor cells [22, 36, 59], and hence, it seems that glioma cells need the stimulus mediated by the neuropeptide, and for this reason cancer cells, including glioma, overexpress the NK-1R [24, 43, 60]. Blocking the stimulus derived from ligand-receptor (SP/NK-1R) activation (by antagonists or silencing NK-1R expression) can trigger apoptotic mechanism in glioma cells.

We have also demonstrated that the *TAC1* silencing by siRNA did not produce any change in GAMG glioma cells and, hence, the absence of SP synthesis in these cells did not affect their survival. SP is not crucial for glioma cells, since the peptide can be synthesized by immune cells (tumor microenvironment) and/or by nerve cells. SP can also reach glioma cells from the blood, and/or the activation of the NK-1R located in glioma cells can be carried out by other peptides belonging to the tachykinin family (e.g., hemokinin-1 (HK-1)). In fact, it is known that HK-1 facilitates the proliferation of glioma cells [19]. This finding highlights the importance of the NK-1R in glioma cells because there are at least two peptides, SP and HK-1, that can activate this receptor. Thus, the crucial point is the expression of the NK-1R (overexpressed in cancer cells), because after the binding of SP to this receptor, all the produced effects are beneficial for human tumor cells: antiapoptotic effect, mitogenesis, facilitation of cell migration, and increased transcription of NK-1R and Warburg effect [5, 38].

As cancer cells overexpress the NK-1R, two different therapeutic strategies could be used not only against glioma but against any tumor type: pharmacological, using NK-1R antagonists [26, 47, 48, 61, 62], and/or genetic by applying the *TAC1R* siRNA method [3, 15]. Both strategies could improve the prognosis and survival of patients suffering from cancer, including glioma, because, in absence of the NK-1R, glioma cells can die by apoptosis as a consequence of starvation.

## 5. Conclusions

To summarize, the presence of both SP and the NK-1R has been shown in the nucleus (full-length isoform) and cytoplasm (truncated isoform) of human glioma cells. These data suggest that the peptide could exert intracrine (nucleocrine), autocrine, paracrine and/or endocrine actions. It has also been demonstrated that the NK-1R is necessary for the survival of human GAMG and U87 MG glioma cells, but the expression of the *TAC1* gene is not needed for the viability of these cells. In contrast, the NK-1R is not essential for the survival of the human nontumor normal fibroblast cells. Our study increases knowledge about involvement of the SP/NK-1R system in glioma and also confirms that the NK1-R is a promising and specific therapeutic target (acting like an Achilles heel) for the treatment of glioma. In addition, SP mediates a common mechanism for the proliferation of cancer cells, and the overexpression of the NK-1R in these cells opens up the possibility for a specific therapeutic treatment against any type of tumor.

## Data Availability

The data that support the findings of this study are available from corresponding authors upon reasonable request.

## Supplementary Materials

Supplementary 1 Negative control to analyze the level of nonspecific background signal produced by rabbit primary antibodies. Primary antibodies were omitted and replaced by nonimmune serum. Antirabbit secondary antibody conjugated to Alexa Fluor 658 (red) (A and B) or Alexa Fluor 488 (green) (C and D) was used and nuclei (Blue) were stained by Hoechst. Scale bar =50 *μ*m.

Supplementary 2 NK-1R expression in siTAC1R U-87 glioma cells. (A) Images from glioma cultures at 6 h with 5 nM of siTAC1R. (B) NK-1R and *β*-actin immunoblot. (C) Immunoblot analysis after measuring the OD bands relative to *β*-actin. Data are shown as the mean ± SEM of three independent experiments (*n* = 3 per group). (D) Cell counting after 6 h of treatment was performed as described in Materials and Methods.
