# Supplementary figures and images for: The Neurokinin-1 Receptor Is Essential for the Viability of Human Glioma Cells: A Possible Target for Treating Glioblastoma
Source: Biomed Res Int. 2022 Apr 4;2022:6291504. doi: 10.1155/2022/6291504 (PMC9006081; doi:10.1155/2022/6291504)

## Slide 1
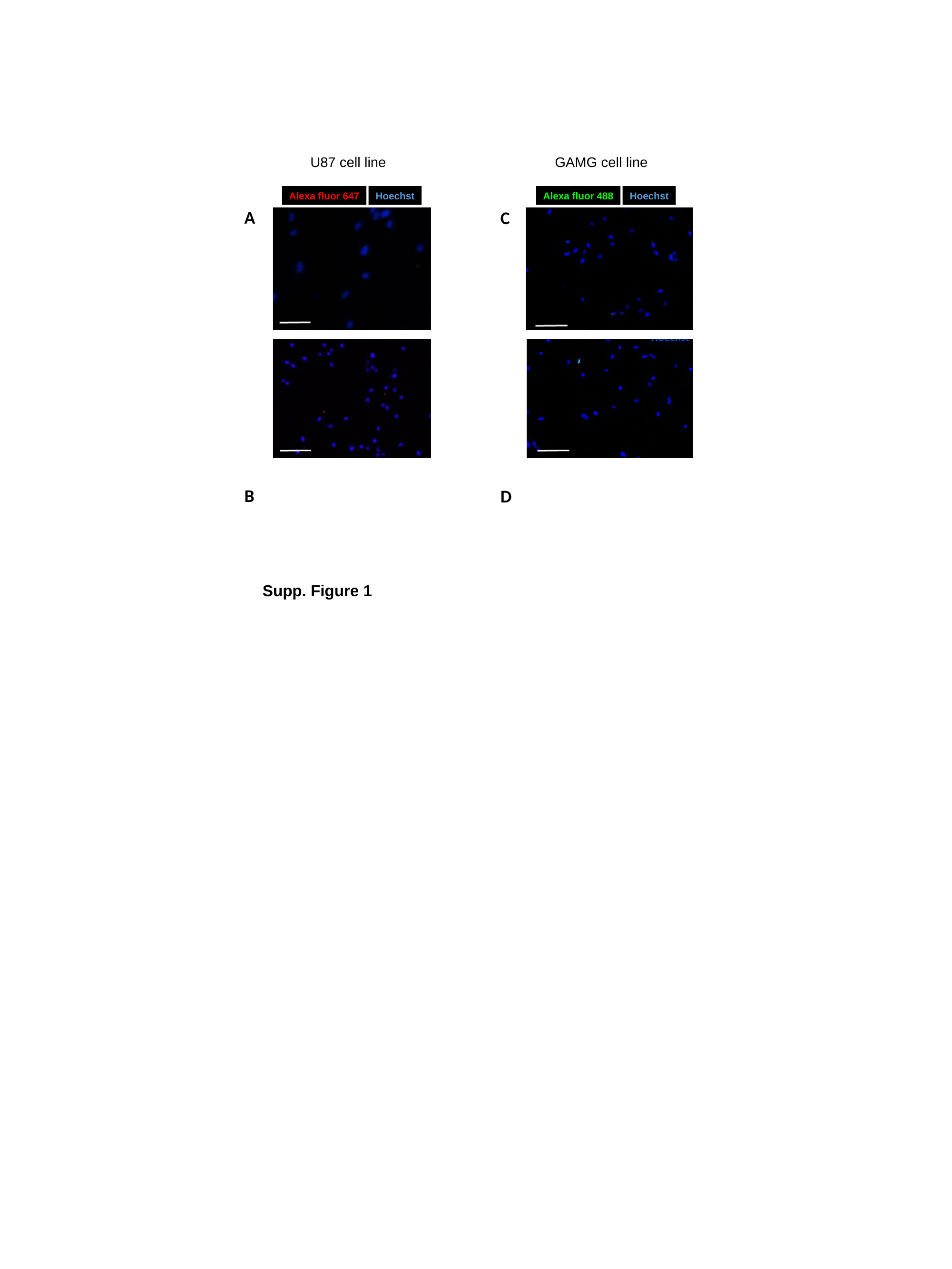

U87 cell line
GAMG cell line
Alexa fluor 647
Hoechst
Alexa fluor 488
Hoechst
A
B
C
D
Supp. Figure 1

Supplement: Supplementary 1 — Negative control to analyze the level of nonspecific background signal produced by rabbit primary antibodies. Primary antibodies were omitted and replaced by nonimmune serum. Antirabbit secondary antibody conjugated to Alexa Fluor 658 (red) (A and B) or Alexa Fluor 488 (green) (C and D) was used and nuclei (Blue) were stained by Hoechst. Scale bar =50 μm. [file 6291504.f1.pptx]
