# Supplementary material for: The Neurokinin-1 Receptor Is Essential for the Viability of Human Glioma Cells: A Possible Target for Treating Glioblastoma
Source: Biomed Res Int. 2022 Apr 4;2022:6291504. doi: 10.1155/2022/6291504 (PMC9006081; doi:10.1155/2022/6291504)
Supplement: Supplementary 2 — NK-1R expression in siTAC1R U-87 glioma cells. (A) Images from glioma cultures at 6 h with 5 nM of siTAC1R. (B) NK-1R and β-actin immunoblot. (C) Immunoblot analysis after measuring the OD bands relative to β-actin. Data are shown as the mean ± SEM of three independent experiments (n = 3 per group). (D) Cell counting after 6 h of treatment was performed as described in Materials and Methods. [file 6291504.f2.pptx]

## Slide 1
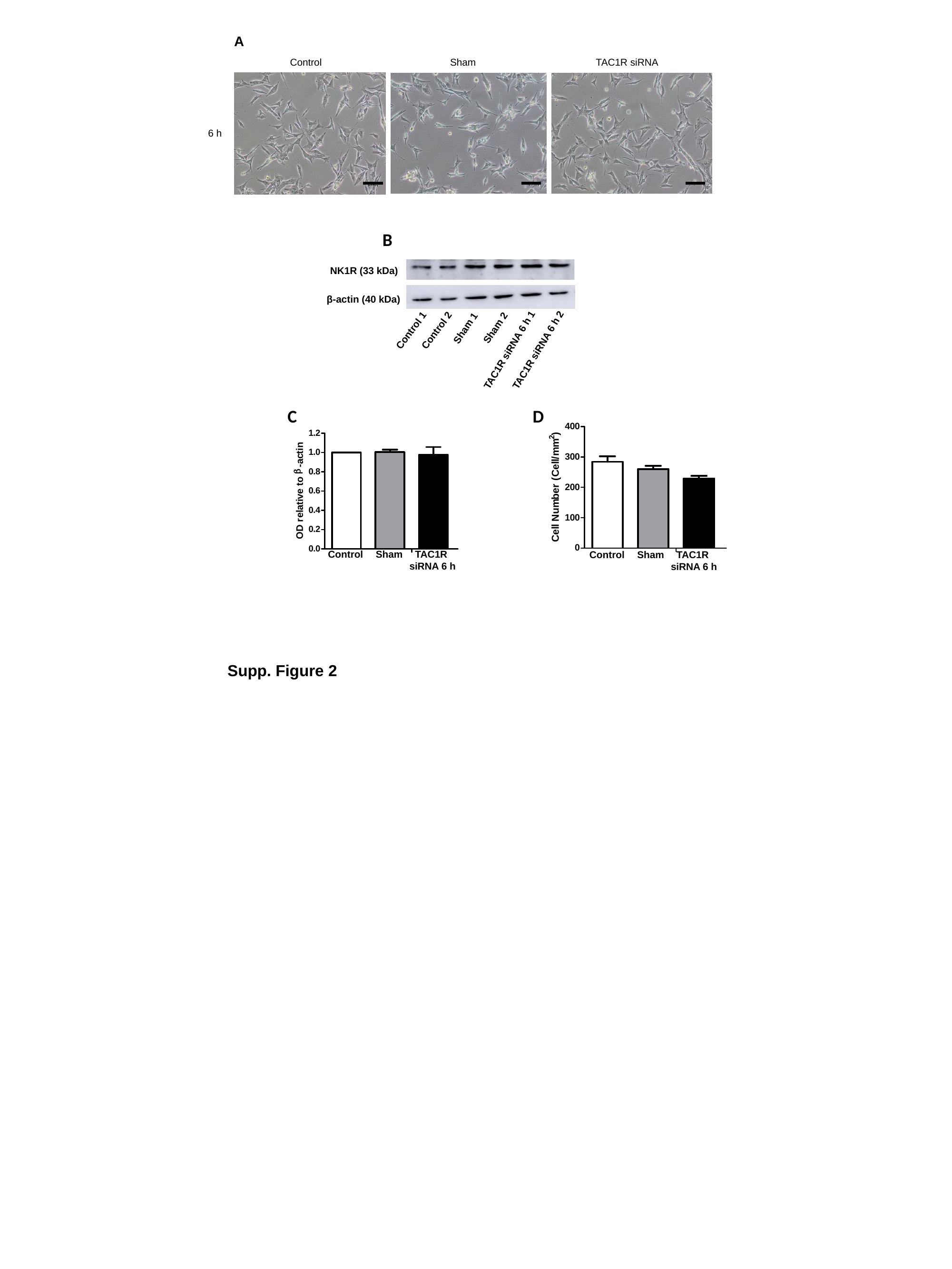

A
Control Sham TAC1R siRNA
6 h
B
Sham 2
Sham 1
Control 1
Control 2
TAC1R siRNA 6 h 1
TAC1R siRNA 6 h 2
NK1R (33 kDa)
β-actin (40 kDa)
C
D
Control
Sham
TAC1R
siRNA 6 h
Control
Sham
TAC1R
siRNA 6 h
Supp. Figure 2
